# Benefits of Intensive Rehabilitation Programs Using Physiotherapeutic Scoliosis-Specific Exercises in Adolescent Idiopathic Scoliosis

**DOI:** 10.7759/cureus.76359

**Published:** 2024-12-25

**Authors:** Jean-François Catanzariti, Fabien Moretto, Quentin Hanot, Chloé Adam, Gemma Renaud, Anthony Brouillard

**Affiliations:** 1 Research, SMR Pédiatrique Marc Sautelet - APF France Handicap, Villeneuve d'Ascq, FRA; 2 Consultation and Research, La Maison de la Scoliose, Villeneuve d'Ascq, FRA; 3 Hospitalization, SMR pédiatrique Marc Sautelet - APF France Handicap, Villeneuve d'Ascq, FRA; 4 Consultation, La Maison de la Scoliose, Villeneuve d'Ascq, FRA; 5 Physiotherapy, Institut de Kinésithérapie Pédicurie Podologie Orthopédie, Lille, FRA; 6 Research, SMR pédiatrique Marc Sautelet - APF France Handicap, Villeneuve d'Ascq, FRA; 7 Sports Medicine, La Maison de la Scoliose, Villeneuve d'Ascq, FRA

**Keywords:** adolescent, idiopathic scoliosis, intensive rehabilitation, multidisciplinary team, physiotherapeutic scoliosis specific exercises

## Abstract

Purpose

Adolescent Idiopathic Scoliosis (AIS) affects 3% of adolescents. Physiotherapeutic Scoliosis Specific Exercises (PSSE) are recommended to limit AIS progression, especially within intensive multidisciplinary programs. Our study evaluated the efficiency of these programs in AIS cases with a high progression risk.

Methods

We conducted a controlled retrospective observational study using data collected from a multicenter cohort that was prospectively collected. One hundred and forty-three major AIS cases with a high progression risk, treated with a corrective brace, were included and divided into two matched groups. In the PSSE group, 72 adolescents followed an intensive 4-week PSSE rehabilitation program; in the control group, 71 adolescents did not follow this program. Patient files were assessed at V0 (inclusion), V1 (6 to 12 months after V0) and V2 (≥ 6 months after V1). The evaluation criteria were: change in Cobb angle and percentage of patients reaching surgical stage at V2.

Results

At V1, 54.2% of patients in the PSSE group showed improvement compared to 16.9% in the control group (p < 0.001). In contrast, 38.9% of patients in the PSSE group were stabilized, compared to 53.3% in the control group (p = 0.2).

At V2, 34.7% of patients in the PSSE group improved compared to 15.5.% in the control group (p <0.006). At V2, 55.6% of patients in the PSSE group were stabilised versus 40.8% in the control group (p < 0.05). At V2, 8.3% of patients in the PSSE group reached the surgical stage versus 21.1% in the control group (p = 0.005).

Conclusion

Our study is an additional argument in favor of using PSSE rehabilitation in AIS.

## Introduction

Adolescent Idiopathic Scoliosis (AIS) is a three-dimensional spinal deformity affecting about 3% of adolescents aged 10-16 [1,2]. Severe forms can impair quality of life, causing pain, respiratory issues, and psychological challenges [1,2].

AIS patients with immature skeletons (Risser ≤ 3) face higher risks of curve progression. For these patients, if the Cobb angle of their scoliotic curvature reaches or exceeds 20°, wearing a corrective brace is necessary to prevent surgical intervention (arthrodesis) [1,2,3]. Weinstein et al.'s BRAIST study confirmed the brace's effectiveness in slowing progression in patients with a Cobb angle of 20°-40° and skeletal immaturity [2,3]. The BRAIST controlled trial (corrective brace versus observation) demonstrated the significant efficacy of the brace in preventing the progression of scoliotic curvature up to 50°, the surgical threshold [3]. In the observation group, 58% of patients reached the surgical stage, compared to only 28% in the brace group. The authors showed a dose-dependent effect: the longer the brace was worn, the greater its efficiency [3]. Thus, a brace worn for more than 12 hours a day was effective at 90% in preventing the patient from reaching the surgical threshold [3]. Despite BRAIST's findings, subsequent research has reaffirmed the efficacy of wearing a corrective brace in AIS with a high progression risk [4,5]. However, as indicated in a 2015 Cochrane review of the literature, the level of evidence remains low partly due to the difficulties of conducting randomized controlled trials, as many parents refused to randomize their children [6].

The benefits of combining rehabilitation with bracing remain controversial [2]. Rehabilitation aims to enhance the brace's effectiveness and mitigate its side effects, such as peri-vertebral amyotrophy and proprioceptive deficit, respiratory restriction, thoracic hypokyphosis, and stiffening [6-10]. The International Society on Scoliosis Orthopaedic and Rehabilitation Treatment (SOSORT) recommends specific rehabilitation therapy known as PSSE (Physiotherapeutic Scoliosis Specific Exercises), to limit scoliosis progression [7,8]. These personalized rehabilitation exercises are adapted to each patient's scoliosis [7,8]. The aims of PSSE programs are learning and stabilization of the corrected posture, three-dimensional active self-correction of vertebral deformity in daily life, and therapeutic education [7,8]. A recent literature review shows that these PSSE programs, combined with wearing a corrective brace, can improve Cobb angle [8]. To increase the effectiveness of these programs, the SOSORT advocates for intensive, multidisciplinary PSSE programs within specialized centers known as 'Scoliosis Schools' [9,10]. Research on the combined effect of bracing and PSSE for AIS is sparse and limited by short follow-up periods and difficulties in conducting randomized trials in the pediatric population [2,7-10]. 

Therefore, we conducted a retrospective study based on a multicentric cohort of children and adolescents with trunk deformity. Our objectives were to 1) assess the impact of intensive PSSE programs combined with bracing on the Cobb angle and 2) evaluate whether PSSE reduces the risk of surgery.

## Materials and methods

Our study is a retrospective controlled study. The patients were treated in two care centers specialized in treating spinal deformities in children and adolescents. The follow-up was 12 months. The data from our cohort were collected prospectively (authorization number for cohort n°2016-04-05-CIER-GHICL). The study was approved by our research committee (n°2021-06-001-CMS-PR), and informed consent was obtained from patients and guardians, adhering to the Declaration of Helsinki on patient protection. The study included two groups: the PSSE group underwent a 4-week intensive rehabilitation program, while the control group received 30-minute non-specific rehabilitation sessions twice weekly. Between January and December 2021, 143 patient files met inclusion and exclusion criteria. The PSSE group had 72 patients, and the control group had 71. 

Patients in the PSSE group participated in 3-4 hours of therapy daily, 5 days a week, for 4 consecutive weeks. This included both group and individual sessions. In addition to the PSSE program, patients wore a Cheneau-Toulouse-Munster (CTM)-type corrective brace during the rehabilitation period. A multidisciplinary team specializing in AIS (physiatrist, physiotherapist, certified orthotist, occupational therapist, adapted physical activity instructor, and psychologist) provided treatment. PSSE intensive rehabilitation program was tailored to each patient based on the curvature's topography, Cobb angle and the patient's muscular, and respiratory and proprioceptive capacities. The PSSE program aimed to: (1) Increase awareness of the deformity and promote correction using visual and somaesthetic feedback; (2) Stabilize corrected postures during static and dynamic activities; (3) Strengthen paravertebral musculature; (4) Improve respiratory function; (5) Educate patients on spinal ergonomics, physical activity, and brace use. After the intensive PSSE program, patients continued 30-minute PSSE outpatient sessions twice weekly.

The control group also wore a Cheneau-CTM brace and received 30-minute non-specific outpatient rehabilitation sessions twice a week without an intensive PSSE program.

Inclusion criteria were as follows: AIS patients aged 11-15 years, with a Cobb angle ≥ 30° and < 50°, Risser classification ≤ 3, treatment combining corrective Cheneau-CTM brace prescribed ≥ 12 hours daily, 30-minute outpatient rehabilitation sessions twice a week, at least 12 months of radiographic and clinical follow-up, and no objection to research from the patient or his or her legal guardian following clear, fair, and written information. There was an additional criterion specific to the PSSE group: intensive 4-week PSSE program within 3 months of brace use

Exclusion criteria were: secondary scoliosis (malformative, neurological, syndromic, traumatic)

Three evaluations were performed: at baseline (V0), 6-12 months after baseline (V1), and at least 6 months after V1 (V2). Cobb angles were measured by the patient's regular physiatrist.

The following parameters were recorded at V0: BMI, gender, age, type of curvature, Cobb angle, Risser classification. At V1 and V2, only the radiographic Cobb angle was measured.

Outcome measures

We analyzed the changes in the Cobb angle between V0, V1, and V2. Changes were classified as 'improved' (≥ 5°), 'very improved' (≥ 10°), 'stabilized' (variation < 5°), or 'worsened' (increase ≥ 5°). We considered scoliosis reaching ≥ 50° at V2 as the surgical stage, in reference to the BRAIST study [3].

Data analysis

For numerical variables, normality was tested using the Shapiro-Wilk test. If normally distributed, means and standard deviations were reported and groups were compared using Student's t-test. Otherwise, medians and interquartile ranges were used, with comparisons via the Wilcoxon-Mann-Whitney test. For categorical variables, Chi-square or Fisher's exact test was used. A significance level of 5% was set for all analyses. Statistical analysis was performed using IBM SPSS Statistics 26.0 (IBM Corp., Armonk, USA).

## Results

The characteristics of the study population are summarized in Table 1. The total population was 85.3% female and 14.7% male. At V0, the risk of scoliosis progression appeared higher in the control group, as the Risser stage was smaller. However, there were no significant differences between the two groups.

**Table 1:** Baseline population N = number of participants; M ± SD = mean ± Standard Deviation; IQR = interquartile range; BMI = body mass index; Risser = Risser classification (system used in orthopedics to assess the level of skeletal maturity based on the ossification of the iliac crest apophyses); M/F = male/female; V0 = initial assessment; V1 = 6 to 12 months after V0; V2 = at least 6 months after V1; N.S = not significant; PSSE = Physiotherapeutic Scoliosis Specific Exercises For numerical variables, if the data follows a normal distribution, we used Student's t-test. For numerical variables, if the data does not follow a normal distribution, we used the Wilcoxon-Mann-Whitney test. For qualitative variables, we used the Chi-square test.

Variable	Total	PSSE group	Control group	p-value
N	143	72	71	
Age (years) (M ± SD)	13.1 ± 1.1	13.2 ± 1.1	13.1 ± 1.1	N.S
BMI (M ± SD)	19.0 ± 2.8	19.1 ± 2.8	19.0 ± 2.7	N.S
Cobb’s angle (M ± SD)	36.8° ± 6.4	37.4° ± 6.7	36.1° ± 6.0	N.S
Risser med [IQR]	2 [1 ; 2]	2 [1 ; 2]	1 [0 ; 2]	N.S
Gender ratio M/F	1/5.8	1/6.2	1/5.4	N.S
Type of scoliosis				N.S
Single curvature	28.9%	23.9%	33.8%	
Double curvature	61.1%	66.2%	62.0%	
Triple curvature	7.0%	9.9%	4.2%	
Period V0-V1 in months (M ± SD)	7.3 ± 1.3	7.4 ± 1.4	7.1 ± 1.2	N.S
Period V0-V2 in months (M ± SD)	14.5 ± 2.3	14.7 ± 2.6	14.3 ± 2.0	N.S

The main results are presented in the subsequent tables. In the PSSE group, the mean Cobb angle decreased from 37.4° (V0) to 34.9° (V2), while it increased from 36.1° to 41.0° in the control group (p < 0.001) (Table 2).

**Table 2:** Change in mean Cobb angle N = number of participants; M ± SD = mean ± Standard Deviation; V0 = initial assessment; V1 = 6 to 12 months after V0; V2 = at least 6 months after V1 period V0/V1 = period between V0 and V1; period V0/V2 = period between V0 and V2; * = significant result (p < 0.05); N.S = not significant; PSSE: Physiotherapeutic Scoliosis Specific Exercises For the results in Table 2, we used the Student's t-test.

Variable	PSSE group	Control group	p-value
N	72	71	-
Cobb angle at V0: M +/- SD	37.4° +/- 6.7	36.1° +/- 5.9	N.S
Cobb angle at V1: M +/- SD	33.5° +/- 9.0	37.5° +/- 8.9	0.01*
Cobb angle at V2: M +/- SD	34.9° +/- 8.9	41.0° +/- 11.3	< 0.001*
p (period V0/V1)	< 0.001*	0.181	
p ( period V0/V2)	0.034*	< 0.001*	

A decline in efficacy was observed between V1 and V2 in the PSSE group. At V2, 34.7% of PSSE group patients improved, 55.6% stabilized, and 9.7% worsened, while in the control group, 15.5% improved, 40.8% stabilized, and 43.7% worsened (Table 3).

**Table 3:** Percentage of participants showing improvement, stabilization or worsening of Cobb angle V0 = initial assessment; V1 = 6 to 12 months after V0; V2 = at least 6 months after V1 period V0/V1 = period between V0 and V1; period V0/V2 = period between V0 and V2; p* = significant difference; PSSE: Physiotherapeutic Scoliosis Specific Exercises For the results in Table 3, we used the Chi-square test.

Variable	V0/V1			V1/V2			V0/V2		
	PSSE	Control	p-value	PSSE	Control	p-value	PSSE	Control	p-value
N	72	71		72	71		72	71	
Improvement N (%)	39 (54.2%)	12 (16.9%)	<0.001*	08 (11.1%)	07 (9.9%)	0.59	25 (34.7%)	11 (15.5%)	0.006*
Stabilization N (%)	28 (38.9%)	38 (53.5%)	0.2	47 (65.3%)	39 (54.9%)	0.36	40 (55.6%)	29 (40.8%)	<0.05*
Worsening N (%)	5 (6.9%)	21 (29.6%)	0.001*	17 (23.6%)	25 (35.2%)	0.09	07 (9.7%)	31 (43.7%)	0.001*
Surgical stage N (%)							06 (8.3%)	17 (23.9%)	0.005*

Overall, 90.3% of PSSE group patients experienced stabilization or improvement at V2, compared to 56.3% in the control group. Furthermore, 8.3% of patients in the PSSE group and 21.1% in the control group reached the surgical threshold (*p=0.005*) (Table 4).

**Table 4:** Evolution of the mean delta of the Cobb angle between the different periods N = number of participants; V0 = initial assessment; V1 = date of radiographic check-up carried out 6 to 12 months after V0; V2 = date of radiographic check-up carried out at least 6 months after V1; V0/V1 = period between V0 and V1; V0/V2 = period between V0 and V2; V1/V2 = period between V1 and V2; M ± SD = mean ± Standard Deviation; PSSE = Physiotherapeutic Scoliosis Specific Exercises group; Control = Control group; * = significant result (p <0.05); N.S = not significant For the results in Table 4, we used the Student's t-test.

Variable	Total	PSSE	Control	p-value
N	143	72	71	-
Period V0/V1 : M ± SD	-1.3° ± 7.2	-4.2° ± 7.0	+1.7° ± 6.1	<0.001*
Period V1/V2 : M ± SD	+2.6° ± 6.4	+1.8° ± 5.8	+3.5° ± 6.9	N.S
Period V0/V2 : M ± SD	+1.1° ± 9.3	-2.4° ± 8.0	+4.8° ± 9.1	<0.001*

## Discussion

Our study evaluated non-surgical treatment of major AIS cases with a high risk of progression. Our findings suggest that a 4-week intensive PSSE program combined with bracing should be included in the therapeutic approach for high-risk AIS. After 14 months of follow-up, scoliosis was stabilized or improved in 86% of the PSSE group patients versus 49.3% in the control group. Patients in the PSSE group had a significantly lower risk of reaching the surgical stage. However, PSSE’s effectiveness declined over time (V1 to V2), indicating a potential need for periodic "booster" sessions until skeletal maturity. The duration of these booster sessions would have to be evaluated by further studies.

Intensive PSSE rehabilitation programs in AIS have previously been described [7,10,11]. The duration of these intensive rehabilitation programs varied between 2 and 12 weeks. Studies on their effectiveness have been carried out [8,10,11]. They most often involved non-homogeneous populations combining minor and major AIS or without a control group. Few of these studies have examined the impact of this type of therapy on patients with AIS with a high progression risk wearing a corrective brace. These characteristics make comparison with our work difficult. Kwan's study is more closely related to ours [12]. It is a prospective controlled study, carried out on 48 patients with AIS aged between 10 and 15 years, with a Cobb angle between 25° and 40°, and a Risser test between 0 and 2. The average follow-up was 18.1 months. The "treatment" group wore a corrective brace for at least 18 hours a day and participated in an outpatient PSSE rehabilitation program for 8 weeks. The "control" group simply wore a corrective brace for at least 18 hours a day. In the PSSE group, the scoliosis was improved or stabilized in 79% of cases, compared with 50% in the control group, these results are similar to ours. Follow-up time was slightly longer than in our study and the scoliosis was less severe. This difference may account for the variations in outcomes between the two studies.

Our research is based on the retrospective evaluation of a prospective, multicentric, pediatric cohort of patients suffering from trunk deformity, in particular AIS. Theoretically, this method could lead to a selection bias. We took various measures to limit this risk of bias. We defined an inclusion period, evaluating the files of all patients treated consecutively during this time span according to precise inclusion and exclusion criteria. The main risk of bias is to minimize the results in the control group. To verify this, we compared our results with those of the reference BRAIST study by Weinstein et al (Table 5) [3]. This study is the first to validate brace treatment in AIS. It compared a "simple monitoring without treatment" group with a "brace" group. The "brace" group in the BRAIST study corresponds to the control group in our work. In the BRAIST study, 25% of participants in the brace group reached the surgical stage [3]. In our study, 21.1% of participants in the control group reached the surgical stage, i.e. comparable results. Therefore, the choice of our control group does not appear to be biased, since the participants responded equally to wearing a brace as the "brace" group in the BRAIST study [3].

**Table 5:** Comparison of the bracing group in Weinstein et al.'s study and the control group in our study M ± SD = Mean ± Standard Deviation Weinstein et al. [3]

Variable	Bracing Group (Weinstein et al.)	Control group (our study)
Age (years) (M ± SD)	12.7 ± 1.0	13.1 ± 1.1
% Females	92	84.5
Cobb’s angle (degrees) (M ± SD)	30.5 ± 5.8	36.1 ± 6.0
% Nonsurgical stage	75%	78.9%

To definitively address this potential bias, conducting prospective, randomized, multicenter studies would be necessary. Ideally, all the patients included should benefit from monitoring brace-wearing time using built-in sensors and blind radiographic assessment by two independent doctors. It would also be interesting to measure other clinically relevant parameters such as quality of life, angle of trunk rotation, and algo-functional evaluation. However, as Weinstein et al. explain in their article on the BRAIST study, randomization in a pediatric population is difficult [3]. The BRAIST study was initially designed as a randomized trial (brace wearing versus observation) but was transformed into a controlled, non-randomized study due to recruitment difficulties. Indeed, 60% of parents refused randomization. Most of these parents (70%) chose brace treatment for their child, hoping to reduce the risk of surgery. In future randomized trials, presenting intensive PSSE therapy to patients and their parents as a factor likely to reduce the risk of surgery may lead to a high rate of refusal to randomize. Finally, rehabilitation programs, particularly specific ones such as PSSE, are often hard to evaluate by conventional randomized controlled trials, such as those used for drug treatments. Indeed, PSSE programs are personalized for each patient, unlike drug treatments, which are identical for the entire study population. These limitations should be considered in the methodology of future studies.

Our study, with all its limits associated with the use of a cohort, supports the use of intensive, multidisciplinary PSSE programs in AIS cases with a high risk of progression. While our methodology cannot fully explain this positive outcome, we propose the following hypotheses that could serve as topics for future studies.

Therapeutic education for patients and their families by a specialized multidisciplinary team can help improve treatment compliance. Patient-caregiver meetings, supervised by a psychologist and a physiotherapist, create a bond of solidarity between patients. This helps patients to overcome isolation and encourages them to invest in their treatment.

Awareness of the scoliotic deformity through various sensory feedback could limit trunk dysmorphophobia in patients with major AIS [13].

Stabilization of the corrected position in reference to the gravitational vertical takes into account the difficulties of some AIS patients in repositioning their trunk with the vertical [14-16].

Paravertebral muscle strengthening and stimulation of trunk proprioception through physiotherapy and adapted physical activities directly contribute to improving scoliotic curvatures [17].

## Conclusions

In conclusion, our study provides further evidence supporting the use of PSSE rehabilitation in AIS. Additional studies with higher levels of evidence are needed to confirm these findings. However, such studies may be challenging to conduct due to logistical and ethical constraints.

## Appendices

Intensive rehabilitation PSSE protocol

The aims of this intensive PSSE rehabilitation therapy were: (1) awareness of deformity and corrected three-dimensional position, using visual (mirror, webcam) and somaesthetic (therapist's touch, external supports...) feedback; (2) stabilization of the corrected static position (standing, sitting) and during dynamic activities (walking, physical activities... ), using sensory-motor facilitation techniques; (3) improving the performance of paravertebral musculature; (4) improving respiratory function; (5) therapeutic education of AIS patients, spinal ergonomics, regular physical activity and wearing a brace.

PSSE programs are personalized for each patient depending on topography and angle of scoliosis; sagittal plane deformity; physical and psychological abilities; level of knowledge of the deformity and involvement in the therapy; associated co-morbidities, etc. The following protocol describes the techniques used to achieve the aims of intensive PSSE therapy. However, it is not possible to describe each exercise as it is adapted to each patient.

Initial Evaluation

On admission, every patient undergoes a full assessment carried out by various health professionals:

Specialized rehabilitation doctor: general medical examination and background information; treatment already provided; assessment of contraindications to certain physical activities. The doctor also evaluates the patient's and parents' knowledge of AIS. He then explains to them in clear, comprehensible terms the pathophysiology of AIS, the goals of the treatments (corrective brace, PSSE program), and the details of the program (weekday hospitalization, multidisciplinary treatment).

Physiotherapist: pain assessment; knowledge of the patient's deformity in particular topography, angle of trunk rotation (ATR), flat back; passive and active ability to reduce deformity; paravertebral muscle capacity; extensibility of muscular chains; respiratory capacity.

Occupational therapist: evaluation of the sitting position and knowledge of spinal ergonomics.

Example of a Typical Week

- Monday

Physiotherapy: 2x 60 mins

Adapted physical activities: 2x 60 mins

- Tuesday

Physiotherapy: 1 x 60 mins

Adapted physical activities: 1x 60 mins

Psychologist group session: 1x 60 mins

- Wednesday

Physiotherapy: 1 x 60 mins

Adapted physical activities: 1x 60 mins

Occupational therapy: 1x 60 mins

- Thursday

Physiotherapy: 2x 60 mins

Adapted physical activities: 2x 60 mins

- Friday

Physiotherapy: 2x 45 mins

Adapted physical activities: 1x 60 mins
